# Supplementary material for: Deep learning-based classification of speech disorder in stroke and hearing impairment
Source: PLoS One. 2025 May 28;20(5):e0315286. doi: 10.1371/journal.pone.0315286 (PMC12118888; doi:10.1371/journal.pone.0315286)
Supplement: Attachment — Submitted filename: Response to Reviewers.docx [file pone.0315286.s002.docx]

Dear Editor and Reviewers,

We sincerely thank you for your thoughtful and constructive comments on our manuscript. We have carefully addressed each point raised in your reviews and made the necessary revisions to improve the manuscript. Below is a detailed point-by-point response to the reviewers' comments, outlining the changes made in the revised version of the manuscript.

Thank you again for your valuable feedback, which has significantly contributed to enhancing the quality of our work.

Journal requirements:

When submitting your revision, we need you to address these additional requirements.

1. Please ensure that your manuscript meets PLOS ONE's style requirements, including those for file naming.

Response: We have carefully ensured that the manuscript meets PLOS ONE's style requirements, including file naming conventions

2. Please note that PLOS ONE has specific guidelines on code sharing for submissions in which author-generated code underpins the findings in the manuscript. In these cases, all author-generated code must be made available without restrictions upon publication of the work. Please review our guidelines at https://journals.plos.org/plosone/s/materials-and-software-sharing#loc-sharing-code and ensure that your code is shared in a way that follows best practice and facilitates reproducibility and reuse.

Response: We acknowledge the guidelines on code sharing and will follow the recommended practices for code availability as outlined.

3. Thank you for stating the following financial disclosure:

“This work was supported by the Technology Innovation Program(or Industrial Strategic Technology Development Program(K_G012001185601, Building Data Sets for Artificial Intelligence Learning) funded By the Ministry of Trade Industry & Energy(MOTIE, Korea), and by the GRRC program of Gyeonggi province.  [GRRC-Gachon2023(B01), Development of AI-based medical imaging technology]”

Please state what role the funders took in the study.  If the funders had no role, please state: "The funders had no role in study design, data collection and analysis, decision to publish, or preparation of the manuscript."

If this statement is not correct you must amend it as needed.

Please include this amended Role of Funder statement in your cover letter; we will change the online submission form on your behalf.

Response: We have included the statement, 'The funders had no role in study design, data collection and analysis, decision to publish, or preparation of the manuscript,' in the cover letter as requested.

4. When completing the data availability statement of the submission form, you indicated that you will make your data available on acceptance. We strongly recommend all authors decide on a data sharing plan before acceptance, as the process can be lengthy and hold up publication timelines. Please note that, though access restrictions are acceptable now, your entire data will need to be made freely accessible if your manuscript is accepted for publication. This policy applies to all data except where public deposition would breach compliance with the protocol approved by your research ethics board. If you are unable to adhere to our open data policy, please kindly revise your statement to explain your reasoning and we will seek the editor's input on an exemption. Please be assured that, once you have provided your new statement, the assessment of your exemption will not hold up the peer review process.

Response: We confirm our intention to make the data freely accessible upon acceptance of the manuscript.

5. Please amend either the title on the online submission form (via Edit Submission) or the title in the manuscript so that they are identical.

Response: The title has been amended to be consistent across the manuscript and submission form.

6. Please review your reference list to ensure that it is complete and correct. If you have cited papers that have been retracted, please include the rationale for doing so in the manuscript text, or remove these references and replace them with relevant current references. Any changes to the reference list should be mentioned in the rebuttal letter that accompanies your revised manuscript. If you need to cite a retracted article, indicate the article’s retracted status in the References list and also include a citation and full reference for the retraction notice.

Response: We have reviewed the reference list to ensure it is complete and correct, and there are no retracted papers cited.

---------------------------------------------------------------------------------------------------------------------------

Reviewers' comments:

Reviewer's Responses to Questions

**Comments to the Author**

3. Have the authors made all data underlying the findings in their manuscript fully available?

The [PLOS Data policy](http://www.plosone.org/static/policies.action#sharing) requires authors to make all data underlying the findings described in their manuscript fully available without restriction, with rare exception (please refer to the Data Availability Statement in the manuscript PDF file). The data should be provided as part of the manuscript or its supporting information, or deposited to a public repository. For example, in addition to summary statistics, the data points behind means, medians and variance measures should be available. If there are restrictions on publicly sharing data—e.g. participant privacy or use of data from a third party—those must be specified.

Reviewer #1: No

Response: The data underlying the results presented in the study are available from the AIHub database (https://www.aihub.or.kr). To access the data, users must register for a free account on AIHub. All relevant data, including the data points behind the reported means and variance measures, can be downloaded from this repository without restrictions. However, for researchers located outside of South Korea, accessing the data requires a separate agreement with the Korea Information Society Agency (NIA) as per the AIHub data usage policy.

Reviewer #2: Yes

5. Review Comments to the Author

Reviewer #1: This is a well-written manuscript about using deep learning to classify speech disorders in individuals with stroke and hearing impairment. I have some suggestions to improve this manuscript.

Response: We sincerely appreciate your positive evaluation and valuable feedback.

Materials and Methods
Please add details about the ground truth for this study.

Response: We have expanded the "Materials and Methods" section to include detailed information on the ground truth. We specified how the ground truth labels were assigned based on clinical diagnoses confirmed by certified neurologists and audiologists, and described the multi-step verification process by independent speech-language pathologists to ensure label accuracy.

Results
I believe this study would be more compelling if the authors included the confusion matrix results for each model.

Response: We have added confusion matrix results for each model in the Results section.

Discussion
Please include a discussion on why each model achieved different accuracy levels in classifying this type of data.

Response: We have expanded the Discussion section to provide a detailed analysis of why each model performed differently, highlighting the architectural strengths and limitations of ResNet-18, SEResNeXt-18, and Inception V3 in the context of speech disorder classification. Additionally, we have included a comparison of parameter counts across models, as this factor also likely contributed to the observed performance differences.

Additionally, expand on the clinical applications of this system in practice.

Response: We have added a section in the Discussion that outlines potential clinical applications of the proposed classification system, emphasizing its use for early diagnosis, rehabilitation monitoring, and remote risk assessment for stroke and hearing impairment patients.

Reviewer #2: Introduction
The authors highlight the need for this work by presenting the advantages of developing classification-based artificial intelligence models to support the diagnosis of speech disorders. The theoretical foundation of existing studies is outlined, along with the present gaps in the research.
The objective of the study is clearly stated and aligns with the title of the work.

Response: We sincerely appreciate your positive evaluation and valuable feedback.

Materials and Methods
It is recommended to specify the type of study conducted to clarify for the reader that it is retrospective.

Response: We have revised the "Materials and Methods" section to specify that this study was designed as a retrospective analysis using pre-existing clinical voice data.

The specifications of the equipment used are provided, as well as the Python libraries and statistical analysis tools employed.
In addition, the characteristics of the audio recordings are detailed, along with the approval by the Seoul Hospital board regarding the use of these primary source recordings.
The characteristics of the population and sample size are presented.
The procedure for recording the audio samples is described, including the details of the scripts used and the physical conditions during the recordings. A flow diagram is included to enhance the reader's comprehension.
Furthermore, the inclusion and exclusion criteria for the recordings, as well as the total number of recordings used, are outlined. The 8:1:1 ratio used for model development, testing, and validation in the machine learning system is also presented.
The methodological rigor in the development of the AI model, as well as the data selection and classification processes, is evident.

Results
The statistical results of the models are presented, demonstrating very good performance under the evaluated conditions. One model outperformed the other two presented.
Relevant statistical techniques are used to compare the models and analyze data classification.

Discussion
The discussion emphasizes important aspects such as the use of a larger dataset compared to previous studies. Accurate classification of speech disorders associated with stroke and hearing impairment is achieved, rather than merely distinguishing between normal and abnormal speakers. The analysis is based on complete scripts, allowing for a more comprehensive examination of speech patterns and characteristics, contrasting with previous studies that focused on phonation of the vowel /a/.
The discussion also highlights the advantages of analyzing features like pitch breaks, vocal fatigue, and voice breaks, which are expected to enhance the accuracy and flexibility of diagnoses.
The study's limitations primarily concern its focus on voice data in Korean and its restriction to stroke and hearing impairment, without considering other speech disorders.

Response: We sincerely thank you for your thoughtful and encouraging assessment and feedback.
